# Systematic review on measurement properties of questionnaires assessing the neighbourhood environment in the context of youth physical activity behaviour

**DOI:** 10.1186/1471-2458-13-461

**Published:** 2013-05-11

**Authors:** Anne K Reimers, Filip Mess, Jens Bucksch, Darko Jekauc, Alexander Woll

**Affiliations:** 1 Department of Sport Science, University of Konstanz, Konstance, Germany; 2 Institute of Sport and Sport Sciences, Karlsruhe Institute of Technology, Karlsruhe, Germany; 3 School of Public Health, WHO Collaborating Centre for Child and Adolescent Health Promotion, Bielefeld University, Bielefeld, Germany

## Abstract

**Background:**

High-quality measurement instruments for assessing the neighbourhood environment are a prerequisite for identifying associations between the neighbourhood environment and a person’s physical activity. The aim of this systematic review was to identify reliable and valid questionnaires assessing neighbourhood environmental attributes in the context of physical activity behaviours in children and adolescents. In addition, current gaps and best practice models in instrumentation and their evaluation are discussed.

**Methods:**

We conducted a systematic literature search using six databases (Web of Science, Medline, TRID, SportDISCUS, PsycARTICLES and PsycINFO). Two independent reviewers screened the identified English-language peer-reviewed journal articles. Only studies examining the measurement properties of self- or proxy-report questionnaires on any aspects of the neighbourhood environment in children and adolescents aged 3 to 18 years were included. The methodological quality of the included studies was assessed using the COSMIN checklists.

**Results:**

We identified 13 questionnaires on attributes of the neighbourhood environment. Most of these studies were conducted in the United States (n = 7). Eight studies evaluated self-report measures, two studies evaluated parent-report measures and three studies included both administration types. While eight studies had poor methodological quality, we identified three questionnaires with substantial test-retest reliability and two questionnaires with acceptable convergent validity based on sufficient evidential basis.

**Conclusions:**

Based on the results of this review, we recommend that cross-culturally adapted questionnaires should be used and that existing questionnaires should be evaluated especially in diverse samples and in countries other than the United States. Further, high-quality studies on measurement properties should be promoted and measurement models (formative vs. reflexive) should be specified to ensure that appropriate methods for psychometric testing are applied in future studies.

## Background

Physical activity has been associated with several health benefits in children and adolescents [1,2]. However, many children and adolescents in developed countries do not complete the recommended level of at least 60 minutes of moderate to vigorous physical activity every day [3,4]. It has been suggested that environmental aspects in addition to the well-established biological, demographic, psychological, social and behavioural aspects are relevant for physical activity [5,6]. Including changes in the neighbourhood environment in physical activity promotion programs may have a lasting impact on entire populations rather than only a short-term impact on individuals [6,7]. Thus, implementing multilevel interventions may increase physical activity in children and adolescents [8,9].

According to ecological models [8,10], human development and human behaviour are shaped by an interaction between the individuals and their environment. Besides homes, schools and childcare centres, the neighbourhood is a relevant place where children’s and adolescents’ physical activity takes place. Galster broadly defined the neighbourhood as “the bundle of spatially based attributes associated with clusters of residences, sometimes in conjunction with other land uses” [11]. The neighbourhood environment comprises several features and represents presence and quality aspects of important environmental characteristics [12-15]. The effects of the neighbourhood environment on physical activity behaviour are assumed to be context- and behaviour-specific [7].

To date, both objective and subjective instruments of the neighbourhood environment have been employed [16]. The emergence of Geographic Information Systems technology allowed for advances in objective measurement tools [17]. Nevertheless, developing and implementing subjective measurement methods remains indispensable especially because, for instance, self- and proxy-report questionnaires are less costly in large-scale studies. In addition, the perceived environment may be more directly related to a person’s behaviour than objectively measurable environmental attributes [18].

Hence, high-quality subjective measurement methods are a prerequisite for understanding associations between environmental attributes and physical activity behaviours [19] particularly in children and adolescents. Moreover, appropriate conclusions about the measurement properties of instruments can only be drawn from high-quality evaluation studies. However, because many existing instruments are not well-known or well-established, different researchers have simultaneously developed similar instruments. Therefore, a better visibility of existing instruments is required to avoid needless replication of measures and to identify high quality measures and current gaps in instrumentation [20].

Although Brownson and colleagues [19] provided the first comprehensive review of existing measures of the built environment, this review was not based on a systematic literature search and provides limited information about the instruments. In addition, only 4 of 19 included instruments had been tested in youth populations, and this review did not consider the methodological quality of the primary studies. Hence, the methodological quality of the reviewed instruments remains unclear.

The aim of our systematic review was to present self- and proxy-report questionnaires that assess the perceived neighbourhood environment as a predictor of children’s and adolescents’ physical activity behaviours and to identify reliable and valid questionnaires. In addition, current gaps and best practice models in instrumentation and their evaluation are presented.

## Methods

The systematic review was conducted according to the PRISMA guidelines [21].

### Search strategy

In this review, we considered all neighbourhood environmental attributes potentially relating to physical activity behaviours in children and adolescents including aspects such as accessibility, safety, convenience, attractiveness and distances to physical activity and recreational facilities or areas, aspects of urban design and traffic features [12,15].

We conducted a systematic computerized literature search to identify all relevant articles on measurement properties of self- and proxy-report questionnaires concerning the perceived physical environment as a predictor of physical activity behaviour in children and adolescents. On July 4th 2012, we searched the following relevant electronic databases for English-language peer-reviewed journal articles: in *topics* in Web of Science (limits: English language, articles) and Medline (limits: Journal articles), in *keywords* in TRID (Transportation Research International Documentation; limits: English language) and in *abstracts* in SportDISCUS, PsycARTICLES and PsycINFO. The search term consisted of five types of related terms:

1. Construct related terms: environment* OR walkabil* OR neighbourhood

2. Age related terms: adolescen* OR youth OR child* OR girl* OR boy*

3. Outcome related terms: physical activ* OR sport* OR exercise OR walking OR active commut* OR active transport* OR cycl* OR bicycl*

4. Method related terms: instrument* OR measur* OR question* OR scale* OR assess* OR survey

5. Quality assessment terms: valid* OR reliab* OR evaluat* OR psychometric*

At least one term of every term type had to be met. In accordance with recommendations for systematic reviews on measurement properties [22], we screened reference lists and citations of included articles to identify additional relevant studies.

### Eligibility criteria

We used the following eligibility criteria: (i) The study evaluated an instrument on any aspects of the neighbourhood environment related to physical activity behaviour also including active transport, walking or cycling; (ii) the instrument was a self- or proxy-report questionnaire for assessment of the neighbourhood environment on the level of individuals; (iii) the main aim of the study was to evaluate at least one measurement property of the questionnaire, and information on measurement properties were collected intentionally; and (iv) the age range of subjects was 3 to 18 years or their mean age was within this range.

### Selection process

Two independent reviewers (AKR, FM) conducted the stepwise literature search. First, all articles were screened based on titles. In a second step, abstracts of potentially relevant articles were reviewed. If the abstract indicated that the study fulfilled the eligibility criteria or the abstract did not provide sufficient information for selection decision, both reviewers assessed the full texts of articles for eligibility. If necessary, supplementary files were also reviewed for additional information. Discrepancies between article selections were resolved after discussion at the end of the selection process. Additionally, both reviewers screened all reference lists and citations of included articles listed in Web of Science using the same procedure. In one case, the corresponding author of the reviewed article was contacted to request additional information on general characteristics of the instrument that were necessary for selection decision.

### Data extraction

We extracted the target data from the full text articles and from webpages specified therein, from additional files and, if necessary, from related publications. The extracted data included: general characteristics of the instruments, characteristics of the studies and study populations, methodological quality and the results of the studies on measurement properties.

### Methodological quality assessment

Two reviewers (AKR, FM) conducted independent methodological quality assessments to determine the evidential basis of the included studies. We adapted the COSMIN (COnsensus-based Standards for the selection of health Measurement INstruments) [23] checklist–a standardized tool for evaluating the methodological quality of studies on measurement properties of health-related patient-reported outcome measures. We used the taxonomy, terminology, and definitions of measurement properties for health-related patient-reported outcomes defined in the Delphi study within the COSMIN study [24]. The COSMIN checklists considered internal consistency, reliability, measurement error, content validity, structural validity, hypotheses testing, cross-cultural validity, criterion validity and responsiveness. We rated the quality of each measurement property of a study according to the items of the corresponding adapted COSMIN checklist. Only measurement properties of the COSMIN checklists were rated. The four response options in the COSMIN scoring system with the ‘worst score counts’ algorithm [25] did not reveal any differences in the methodological quality between studies. Therefore, we used dichotomous response options [26] and calculated the percentage of items with positive ratings across each checklist to represent the number of quality criteria fulfilled by each study. Because of different numbers of items for each checkbox, missing an item had a different impact on methodological quality scores (MQS) across measurement properties.

Hypotheses testing was defined as the degree to which the scores of an instrument are consistent with the hypotheses based on the assumption that the instrument validly measures the construct to be measured and is subdivided into convergent, discriminant and discriminative validity [24]. Because the included studies only addressed convergent validity, the more common term ‘convergent validity’ will be used in this paper [27].

In accordance with other authors [28,29], the COSMIN manual suggests that internal consistency and structural validity statistic can only be interpreted if the construct is based on a reflective model [26]. In reflective models, the items are manifested by the construct, and the direction of causality is from the construct to the items [30]. Thus, variation in the construct causes variation in the indicators, the items are interchangeable and the items presumably have a high inter-correlation [28,31]. In comparison, in formative constructs the latent variable is formed and defined by the items and the causality is from the items to the construct [30]. Furthermore, the variation in the construct does not necessarily cause variation in the indicator, the items are not interchangeable and the items are not necessarily correlated with each other [28,31]. Statistical procedures assuming inter-item correlation, such as factor analysis or internal consistency, are not appropriate for expressing measurement properties of questionnaires assessing formative constructs [29,32]. We assume that the neighbourhood environment is a formative construct because attributes of the neighbourhood environment (e.g. accessibility of parks or pavements of streets) form the construct of the neighbourhood environment. Changes in the neighbourhood environment do not necessarily cause changes in all indicators and the items may not be inter-correlated. For instance, changes in accessibility to parks causes changes in the neighbourhood environment but is not necessarily associated with changes in pavements of streets. Furthermore, pavements of streets and accessibility of parks may not be inter-correlated. According to these arguments, estimates of internal consistency and structural validity have not been considered for evaluating questionnaires, and the methodological quality of the studies on internal consistency and structural validity were not rated in this review.

### Quality criteria for measurement properties

#### Reliability

Test-retest reliability and inter-rater reliability were considered as substantial for intra-class coefficients (ICC) above 0.75 [33] or Cohen’s kappa above 0.61 [34].

#### Validity

Criterion validity was assessed by Cohen’s kappa [35], and Cohen’s kappa above 0.61 was considered as substantial criterion validity. Convergent validity was considered as acceptable if at least one significant relationship between neighbourhood environmental and physical activity measures (with a theoretical relationship) was found.

## Results

**Figure 1:**
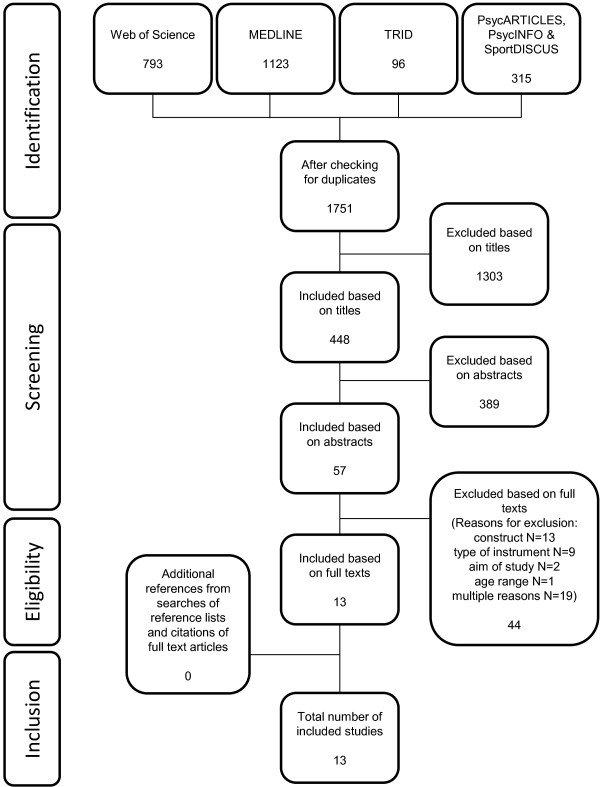
Flow chart of the search and selection process.

The study selection process and reasons for exclusion are summarized in Figure 1. After excluding duplicates, the literature search yielded 1751 articles. After screening titles and abstracts, 57 full text papers were reviewed and 13 articles met the eligibility criteria. Screening reference lists and citations of included articles resulted in no additional articles that met the eligibility criteria. Thus, 13 studies were included in this systematic review.

### Characteristics of included studies

Characteristics of the 13 studies included in this review are presented in Table 1. Seven studies were conducted in the United States [36-42], two in Europe [35,43], two in Australia [44,45], one in Hong Kong [46] and one in Iran [47]. Three studies targeted only female participants [37,40,47]. Eight studies evaluated a self-report measure [37,39,40,42-44,46,47], two a parent-report measure [35,45] and three included both administration forms [36,38,41]. The study samples were recruited mainly from schools (n = 8) [39,40,42-47] or communities (n = 4) [35,36,38,41]. None of the studies explicitly stated the response rates for each measurement property.

**Table 1:** Characteristics of the included studies

**Source**	**n**	**Age**	**Distribution of sex (% male)**	**Administrator**	**Country**	**Setting**	**Sampling**
Dunton et al. [37]	87 47^a^	15.02 ± 0.72	0	adol	USA	intervention study^d^	n.a.
Durant et al. [38]	187^adol^ 171^padol ^116^pchn^	12-18^adol^ 5-11^chn^	40.5^adol^/46.8^adol ^47.8^chn^	adol, p	USA	community	convenience sample
Dwyer et al. [45]	95	3.8 ± 0.74	53	p	Australia	school, hospital, university	convenience sample
Erwin [39]	64	10.27 ± 0.74	31	chn	USA	elementary school	cluster sample
Evenson et al. [40]	610 480^b^	10-15	0	adol	USA	school	cluster sample
Forman et al. [41]	187^adol^ 287^p^ 162^a^	12.0 ± 3.6	48	adol, p	USA	community	convenience sample
		14.7 ± 1.7					
Huang et al. [46]	303 160^b^	11.1 ± 0.9	47.2	chn	Hong Kong	primary school	cluster sample
Hume et al. [44]	39	11.1 ± 0.7	54	chn	Australia	school class	cluster sample
McMinn et al. [35]	24^c^	4	70.8^c^	p	GB	community	n.a.
	389		49.8				
Norman et al. [42]	76	13 ±1.1	45	adol	USA	after school programms	convenience sample
Ommundsen et al. [43]	3958	9.65 ± 0.42^chn^	47.4	chn, adol	Norway, Denmark, Portugal, Estonia	school	two stage cluster sample
		15.49 ± 0.50^adol^					
Pirasteh et al. [47]	545 93^b^	15.74 ± 0.77	0	adol	Iran	school	cluster sample
Rosenberg et al. [36]	171^adol^ 171^padol, a ^116^pchn^	14.6^adol^ 8.3^chn^	49.3^adol^ 47.8^chn^	adol, p	USA	community	convenience sample

Note: adol, adolescents; chn, children; p, parents; padol, parents of adolescents; pchn, parents of children; a Inter-Rater Reliability; b Test-Retest Reliability; c criterion validity study; d adolescents minimally active.

### General characteristics of reviewed instruments

General characteristics of the instruments included in our systematic review are presented in Table 2. The target populations ranged from preschool children to adolescents. The number of items ranged from 4 [42,47] to 66 [36]. Only some questionnaires focused on specific physical activity behaviours, one instrument examined environmental and safety barriers in relation to physical activity in parks and streets [38], one focused on active transport to school [40], one on walking and cycling to three different neighbourhood destinations [41], and one on walking in the neighbourhood [36]. Two studies evaluated the same environment scale originally used in the Amherst Health and Activity Study in English [42] and translated into Persian [47]. Most questionnaires employed Likert scales (Table 2).

**Table 2:** Characteristics of the included questionnaires

**Source**	**Name of instrument**	**Target population**	**Dimensions of environmental construct**	**Number of items**	**Response categories**	**Scoring**
Dunton et al. [37]		adolescent girls	availability of community exercise facilities	26	yes – no	sumscore
Durant et al. [38]		youth	1. environmental barriers to PA in local parks	5	4 point Likert scale	
			2. safety barriers to PA in local parks	6	4 point Likert scale	
			3.environmental barriers to PA in neighbourhood streets	5	4 point Likert scale	
			4. safety barriers to PA in neighbourhood streets	5	4 point Likert scale	
Dwyer et al. [45]	Pre-PAQ	preschool-age children	perception of neighbourhood	8	4 point Likert scale	
Erwin [39]	Preadolescent Environmental Access to PA Questionnaire	9- to 12-year-old children	1. neighbourhood environment	9	yes – no	sumscore
			2. convenient facilities	11	yes – no	sumscore
Evenson et al. [40]		adolescent girls	1. safety	8	5 point Likert scale	
			2. aesthetics	4	5 point Likert scale	
			3. facilities near the home	31	5 point Likert scale (3 items), yes – no (28 items)	sumscore for dichotomous items
Forman et al. [41]		youth	1. environmental barriers for walking and cycling to parks	17	4 point Likert scale	average score
			2. environmental barriers for walking and cycling to shops	17	4 point Likert scale	average score
			3. environmental barriers for walking and cycling to school	17	4 point Likert scale	average score
Huang et al. [46]		Hong Kong Chinese children	1. safety	5	5 point Likert scale	average score
			2. sports facilities	5	yes – no	sumscore
Hume et al. [41]		children	1. physical environment	15	7 point scale	composite score
			2. aesthetics	9	yes – no	sumscore
			3. safety	5	yes – no	sumscore
McMinn et al. [35]		preschool children	local environment	8	5 point Likert scale	
Norman et al. [42]	^a^	adolescents	environment	4	5 point Likert scale	average score
Ommundsen et al. [43]		young people	1. opportunity	3	3 response options	average score
			2. facility	2	3 response options	average score
			3. licence^b^	2	3 response options	average score
Pirasteh et al. [47]	^a^	Iranian adolescent girls	environment	4	5 point Likert scale	
Rosenberg et al. [36]	NEWS-Y	youth	1. land use mix-diversity	20	6 response options	composite score
			2. pedestrian and automobile traffic safety	7	4 point Likert scale	average score
			3. crime safety	6	4 point Likert scale	average score
			4. aesthetics	3	4 point Likert scale	average score
			5. walking/ cycling facilities	3	4 point Likert scale	average score
			6. street connectivity	3	4 point Likert scale	average score
			7. land use mix-access	6	4 point Likert scale	average score
			8. residential density	4	5 response options	composite score
			9. recreation facilities	14	6 response options	composite score

Note: PA, physical activity; a Items originally from the Amhest Health and Activity Study; b dimension is not an aspect of a physical environmental construct in the proper sense, but was mentioned because it was included in the factorial analysis.

### Results and methodological quality of studies on reliability

Nine articles examined the questionnaires’ internal consistency [35-37,41-44,46,47], ten their test-retest reliability [36,38-42,44-47] and three their inter-rater reliability [36,37,41]. Estimates and MQS of studies on reliability are presented in Table 3. Overall, three studies provided information about the percentage of missing values [39,44,46] and two studies described how missing values were handled [37,47]. The MQS of the test-retest reliability studies ranged from 33 to 70 where 100 was the maximum possible score. The test-retest intervals ranged from 6 to 27 days. The studies on inter-rater reliability [36,37,41] examined the agreement of ratings from adolescents and their parents. All inter-rater reliability studies reached MQS of 29. The main flaw of test-retest reliability studies was not ensuring similar test conditions for both measurements (second survey was sent back with a return envelope) [36,38,41,45,47] and that of inter-rater reliability studies was not ensuring independent measurements (e.g. when participants answered the questionnaires at home) [36,37,41].

**Table 3:** Methodological quality and measurement properties of studies on reliability

**Source**	**Dimensions of environmental construct (number of items)**	**Internal consistency**	**Test-retest reliability**			**Inter-rater reliability**	
		**Results**	**MQS**	**Interval [days]**	**Results**	**MQS**	**Results**
Dunton et al. [37]	availability of community exercise facilities (26)	not assessed			not assessed	29	n.s.
Durant et al. [38]	1. environmental barriers to PA in local parks (5)	α = 0.71 - 0.81	38	27	ICC = 0.48 - 0.58		not assessed
	2. safety barriers to PA in local parks (6)	α = 0.64 - 0.70			ICC = 0.57 - 0.76		
	3. environmental barriers to PA in neighbourhood streets (5)	α = 0.80 - 0.87			ICC = 0.49 - 0.61		
	4. safety barriers to PA in neighbourhood streets (5)	α = 0.67 - 0.76			ICC = 0.63 - 0.67		
Dwyer et al. [45]	perception of neighbourhood (8)	not assessed	33	7-14	*Κ* = 0.60 - 0.90		not assessed
Erwin [39]	1. neighbourhood environment (9)	not assessed	70	7-10	ICC = 0.86		not assessed
	2. convenient facilities (11)				ICC = 0.86		
Evenson et al. [40]	1. safety (8)	not assessed	70	6-24 (M = 12)	*Κ* = 0.37 - 0.52		not assessed
	2. aesthetics (4)				*Κ* = 0.31 - 0.39		
	3. facilities near the home (31)				ICC: 0.78		
Forman et al. [41]	1. environmental barriers for walking and cycling to parks (17)	α = 0.70 - 0.84	50	27	ICC = 0.60 - 0.74		ICC = 0.69 - 0.73
	2. environmental barriers for walking and cycling to shops (17)	α = 0.70 - 0.85			ICC = 0.56 - 0.75	29	ICC = 0.46 - 0.68
	3. environmental barriers for walking and cycling to school (17)	α = 0.70 - 0.86			ICC = 0.60 - 0.81		ICC = 0.73 - 0.78
Huang et al. [46]	1. safety (5)	α = 0.71	70	10	ICC = 0.89		not assessed
	2. sports facilities (5)	not assessed			*Κ* = 0.58 - 0.70		
Hume et al. [44]	1. physical environment (15)	not assessed	60	up to 9	ICC = 0.84		not assessed
	2. aesthetics (9)	α = 0.43			ICC = 0.72		
	3. safety (5)	α = 0.65			ICC = 0.88		
McMinn et al. [35]	local environment (8)	α = 0.52 - 0.62			not assessed		not assessed
Norman et al. [42]	environment (4)	α = 0.24 - 0.67	63	7	ICC = 0.60 - 0.64		not assessed
Ommundsen et al. [43]	1. opportunity (3)	α = 0.44			not assessed		not assessed
	2. facility (2)	r = 0.20					
Pirasteh et al. [47]	environment (4)	α = 0.67	38	15	r = 0.38		not assessed
Rosenberg et al. [36]	1. land use mix-diversity (20)	α = 0.87 - 0.93	50	27	ICC = 0.77 - 0.87	29	ICC = 0.77
	2. pedestrian and automobile traffic safety (7)	α = 0.79 - 0.85			ICC = 0.66 - 0.74		ICC = 0.52
	3. crime safety (6)	α = 0.87 - 0.93			ICC = 0.73 - 0.87		ICC = 0.53
	4. neighbourhood aesthetics (3)	α = 0.75 - 0.86			ICC = 0.60 - 0.75		ICC = 0.44
	5. walking/ cycling facilities (3)	α = 0.79 - 0.89			ICC = 0.66 - 0.79		ICC = 0.59
	6. street connectivity (3)	α = 0.72 - 0.75			ICC = 0.56 - 0.61		ICC = 0.47
	7. land use mix-access (6)	α = 0.72 - 0.84			ICC = 0.56 - 0.73		ICC = 0.57
	8. residential density (4)	α = 0.77 - 0.90			ICC = 0.62 - 0.82		ICC = 0.58
	9. recreation facilities (14)	α = 0.80 - 0.84			ICC = 0.67 - 0.73		ICC = 0.55

Note: PA, physical activity; α, Cronbach’s Alpha; *Κ,* Cohen’s kappa; ICC, Intra-class coefficient; r, correlation coefficient; n.s., not significant.

### Results and methodological quality of studies on validity

Six studies examined the convergent validity [36-38,40,41,43], six studies the structural validity [35,38,41,43,46,47] and one study the criterion validity [35] of the questionnaires. Estimates and MQS of validity studies are presented in Table 4. None of the studies provided the percentage of missing values. Two studies described how missing values were handled [37,47]. One study examined criterion validity by using a telephone interview as a ‘gold standard’ [35] (not shown in table) with a MQS of 50, and an agreement between telephone interview and questionnaire responses between 62.5 and 93.8% (*Κ* = 0.00–0.54) on the item level. Six studies assessed the structural validity using an exploratory factor analysis [13,20,24,33,41,43]. Ommundsen and colleagues [43] also conducted a confirmatory factor analysis to perform a cross-cultural, age and gender validation of the instrument. The MQS of studies on convergent validity ranged from 29 to 71. The relationships with non-specific [36,37,40,43] and behaviour or context-specific physical activity measures [36,38,40,41] were tested. Only one study examined the relationship of neighbourhood environmental attributes with objectively measured physical activity [43]. All other studies used subjective measures of physical activity.

**Table 4:** Methodological quality and measurement properties of studies on validity

**Source**	**Dimensions of environmental construct (number of items)**	**Structural validity**	**Convergent validity**^**a**^	
		**Results**	**MQS**	**Results**
Dunton et al. [37]	availability of community exercise facilities (26)	not assessed	57	reported PA indicators (lifestyle activities, vigorous PA, energy expenditure): n.s.
Durant et al. [38]	1. environmental barriers to PA in local parks (5)	PCA: Support for a two factor solution	43	related to reported PA in parks (SR of adol and PR of adol)
	2. safety barriers to PA in local parks (6)			related to reported PA in parks (PR of adol)
	3. environmental barriers to PA in neighbourhood streets (5)	PCA: Support for a two factor solution		related to reported PA in streets (all administrator groups)
	4. safety barriers to PA in neighbourhood streets (5)			related to reported PA in streets (PR of chn)
Evenson et al. [40]	1. safety (8)	not assessed		safe walk/ jog related to PA, seen by others related to ATS
	2. aesthetics (4)		43	trees, things to look at, garbage related to PA, smells related to ATS
	3. facilities near the home (31)			equipment, trails, number of facilities near home related to PA, number of facilities near home related to ATS
Forman et al. [41]	1. environmental barriers for walking and cycling to parks (17)	PCA: Support for a three factor solution (environment, planning/ psychosocial, safety)	29	subscales environment and planning/ psychosocial related to reported walking or bicycling to the specific destination (except for planning/ psychosocial in PR of chn)
	2. environmental barriers for walking and cycling to shops (17)	PCA: Support for a three factor solution (environment, planning/ psychosocial, safety)		all subscales related to reported walking or bicycling to the specific destination (safety only in PR of chn)
	3. environmental barriers for walking and cycling to school (17)	PCA: Support for a three factor solution (environment, planning/ psychosocial, safety)		all subscales related to reported walking or bicycling to the specific destination (safety only in PR of adol)
Huang et al. [46]	1.safety (5)	EFA: Support for an one factor solution		not assessed
	2. sports facilities (5)	not assessed		not assessed
McMinn et al. [35]	local environment (8)	PCA: Support for a two factor solution		not assessed
Ommundsen et al. [43]	1. opportunity (3)	EFA and CFA: Support for a three factor solution	71	related to reported stages of PA change: F(4, 3689) = 29.43**; low objective measured PA vs. high PA associated with lower opportunity scores: (M = 2.60 vs. 2.65; t = 2.10*)
	2. facility (2)			related to reported stages of PA change: F(4, 3689) = 3.60**; low objective measured PA vs. high PA associated with higher facility scores: (M = 1.47 vs. 1.30; t = -2.33*)
	3. licence^b^ (2)			
Pirasteh et al. [47]	environment (4)	PCA: Support for an one factor solution		not assessed
Rosenberg et al. [36]	1. land use mix-diversity (20)	not assessed	57	related to reported walking to shops and to school in different administrator groups
	2. pedestrian and automobile traffic safety (7)			related to reported being active in parks and walking to parks in different administrator groups
	3. crime safety (6)			related to reported walking to shops (PR of chn, SR of adol) and being active in streets (PR of chn)
	4. aesthetics (3)			related to reported being active in parks (PR of chn), walking to parks (SR of a) and being physically active (PR of adol)
	5. walking/ cycling facilities (3)			related to reported being active in parks and walking to shops, school and parks (different administrator groups)
	6. street connectivity (3)			related to reported being active in parks and walking to shops, school and parks (different administrator groups)
	7. land use mix-access (6)			related to reported being active in parks and walking to shops, school and parks (different administrator groups)
	8. residential density (4)			related to reported being active in parks and walking to school (PR of chn, SR of adol)
	9. recreation facilities (14)			related to reported being active in parks and streets and walking to shops, school and parks (different administrator groups)

Note: * Significant at 0.05; ** Significant at 0.001; PA, physical activity; MVPA, moderate-to-vigorous physical activity; EFA, exploratory factor analysis; PCA, principle components analysis; CFA, confirmatory factor analysis; n.s., not significant; SR, self-report; PR, parent-report; adol, adolescents; chn, children; ATS, active transport to school; a due to the aim of this review article only relationships with physical activity behaviours were stated; b dimension is not an aspect of a neighbourhood environmental construct in the proper sense, but was mentioned, because it was included in factorial analysis.

## Discussion

We systematically reviewed 13 studies on measurement properties of self- and proxy-report questionnaires for assessing neighbourhood environmental correlates of physical activity behaviours in youth and rated their methodological quality using modified versions of the COSMIN checklists.

### General issues on measurement

The evidential basis and thus the generalizability of the results of the studies on measurement properties was low partly because of selective samples, non-reporting of response rates, missing values and handling missing values. The main strengths of most studies were that they reported detailed characteristics of participants and had adequate sample sizes.

Many studies recruited selective samples only from urban areas, and sampling procedures were mainly based on school clusters or community based convenience samples, which might lead to clustered samples within similar neighbourhood environments. As discussed by some authors [45], convenience samples might not cover the entire target populations. However, Ommundsen and colleagues [43] used a two-stage cluster sampling procedure for recruiting a representative sample, and Huang and colleagues [46] recruited a sample with a reasonable representation of families from different socioeconomic status areas. Ommundsen and colleagues [43] examined the measurement properties in international samples (i.e. Norway, Denmark, Portugal and Estonia) and demonstrated cross-cultural invariance of their measure. Most other studies were conducted in the United States, and it is thus unknown if the employed questionnaires are appropriate for study populations outside of the United States. Our results further emphasize the previously stated [16,48] need for international studies on measurement properties and additional evaluation studies conducted outside the United States for examining cross-cultural appropriateness of these measures, improving generalizability and facilitating international comparison of research findings of neighbourhood environmental impacts on physical activity behaviour.

Few of the reviewed studies reported response rates and percentages of missing values and how missing values were handled. Item and unit nonresponse may indicate selection bias and hence limit the generalizability of the results [26,49]. High percentages and inappropriate handling missing values can lead to bias in parameter estimates, lower sample sizes and lower statistical power [50]. Non-random missing items may bias the results and lead to misinterpretation and misjudgement of measurement properties of instruments [26]. In addition, a high percentage of missing values on one item may indicate that this item is not relevant for the target population or that it is ambiguously formulated [23].

Considering the flaws of previous studies, we recommend that future research should accurately report response rates and provide information on handling missing values. In addition, randomness of nonresponse should be examined and reported in future studies.

### Reliability

Four test-retest reliability studies had high methodological quality [39,44,46,51] and three of these studies also yielded substantial reliability estimates on all indices [39,44,46]. Thus, there is sufficient evidence supporting substantial test-retest reliability of the 9-item neighbourhood environment and the 11-item convenient facilities scales of Erwin [39], the 5-item neighbourhood safety and 5-item sports facilities scales of Huang and colleagues [46] and the 15-item physical environment, 9-item aesthetics and 5-item safety scales of Hume and colleagues [44]. These questionnaires are recommended based on their test-retest reliability. Nevertheless, to date the validity of these questionnaires has not been evaluated (except the structural validity in the safety scale of Huang and colleagues [46]) and should be assessed in further studies.

The test-retest intervals of the included studies on test-retest reliability ranged from 6 to 27 days and varied within some of the studies. To the best of our knowledge, to date an appropriate test-retest interval in reliability testing of questionnaires on physical environmental attributes has not been specified. Considering the relative stability of perceived physical environmental constructs such as facilities or street connectivity, potential memory effects should be avoided by choosing sufficiently long test-retest intervals. Moreover, one should consider that the first assessment may have drawn the participant’s attention to specific aspects of their neighbourhood environment and thereby potentially influence the responses of the second assessment [52].

Only three studies examined inter-rater reliability, and the methodological quality scores of these studies were low. Thus, to date no conclusion about acceptability of inter-rater reliability estimates can be drawn. It is possible that the agreement between self- and proxy-responses is an inadequate criterion of measurement quality because the perception of the environment may differ between parent and child [53].

A common flaw of reliability studies is that the questionnaire administration was not supervised by a researcher (e.g. when providing a home administration questionnaire). Unsupervised questionnaire administration leads to uncertainty about the time of administration (test-retest intervals), administrators and test conditions. This issue could be addressed by administering the instrument in the classroom under the supervision of a researcher, choosing a specified test-retest interval (e.g. one week) and conducting the second measurement exactly at the same time of the day as the first measurement.

Vague definitions of the area of interest (e.g. the neighbourhood) may have caused low test-retest and inter-rater reliability estimates in some studies [54]. Administrator responses could be influenced by different interpretations of the neighbourhood and therefore lead to divergent answers because, for instance, the term ‘neighbourhood’ might be associated with various geographical extensions. Therefore, questionnaires should define the exact area of interest. For example, Rosenberg and colleagues [36] and Huang and colleagues [46] defined the neighbourhood as “the local area around [the adolescent’s] home, within a 10–15 minute walk in any direction” and “the area within a 20-minute walk or drive from [the children’s] home”, respectively.

Therefore, we recommend that the neighbourhood should be concisely defined in questionnaires of neighbourhood environmental attributes and the administration of questionnaires should be standardized and supervised.

### Validity

McMinn and colleagues [35] evaluated the criterion validity describing the degree to which the scores of an instrument are an adequate reflection of a ‘gold standard’. In this study, questionnaire responses were compared with interview responses of the same persons, that is interviews including the same items as the questionnaire were conducted with the participants who answered the questionnaire. Kappa estimates of this study were not substantial and did not support criterion validity of the perceived local environment questionnaire. The small number of studies on criterion validity may be related to the lack of a well-established ‘gold standard’ that could be used as a reference for new measures [35]. Objective environmental measures such as GIS data do not adequately reflect the same constructs as subjective environmental measures [18] and thus cannot be accepted as a ‘gold standard’. Several studies have shown low agreement between subjectively and objectively measured features of the physical environment [18,55]. Physically active people may know their neighbourhood better then physically inactive people and hence achieve higher agreement between subjectively and objectively measured aspects of their neighbourhood [56]. The criterion validity study included in this review used an interview method as a ‘gold standard’ because this method enables a greater insight into participants’ answers and provides important feedback. In addition, both types of instrument administrations (questionnaire and interview) capture perceptions of the same target person and this procedure could produce correlated bias of both measurements and is prone to social desirability. The small sample size of the criterion validity study (N = 24), which is presumably related to the high cost and time needed for interview methods, queries the level of evidence of the results [35].

With regards to convergent validity, the studies of Ommundsen and colleagues [43] and Rosenberg and colleagues [36] had high methodological quality scores in combination with acceptable estimates of convergent validity. Therefore, sufficient evidence supports convergent validity of the 3-item opportunity and 2-item facility scales in the study by Ommundsen and colleagues [43] and the 66-items NEWS-Y in the study by Rosenberg and colleagues [36]. Because the participants of the study by Ommunden and colleagues [43] were randomly selected from four European countries, this measure seems to be appropriate for usage in different countries across Europe. Both questionnaires [36,43] are recommended based on their convergent validity. Nevertheless, the reliability of the questionnaires developed by Ommundsen and colleagues [43] should be evaluated in further studies. Although the reliability of the NEWS-Y [36] has been assessed, the methodological quality of these reliability studies was limited. Thus, the reliability of the NEWS-Y should be evaluated in studies with high methodological quality.

Ding and Gebel [16] suggested to focus on conceptually matched associates between environmental attributes and domains of physical activity behaviour, and thus neighbourhood environmental measures should be behaviour- and context-specific [7]. For example, neighbourhood environmental barrier scales were developed to measure behaviour and context-specific environmental attributes such as barriers for walking and cycling to parks or barriers for physical activity in local parks [38,41]. Common objective methods for assessing physical activity such as accelerometry on their own are not capable of capturing context-specific physical activity behaviours. The use of a more time consuming but feasible log booklet [57] or the ecological momentary assessment [58] are alternatives to these objective measures.

We recommend that convergent validity of instruments should be evaluated by examining theoretically linked neighbourhood environmental constructs and physical activity measures. Specific hypotheses regarding expected correlations with other constructs must be formulated a priori when developing a new measure.

### Structural validity and internal consistency

As described above, we assume that neighbourhood environment is based on formative constructs. Some authors propose formative measurement models in physical activity environment measures [27,59,60], and low internal consistency across studies [35,42-44,47] indicate that the underlying constructs are formative. Consequently, statistical procedures such as calculating Cronbach’s Alpha or factor analyses may not be appropriate for estimating internal consistency and structural validity. However, this issue is only rarely addressed in the scientific literature on physical environmental constructs in relation to physical activity behaviour. The studies on internal consistency and structural validity included in this review did not appropriately consider this issue when evaluating the measurement properties of the questionnaires. Future studies evaluating neighbourhood environmental measures should consider the measurement model of the underlying construct because of its importance for investigating measurement properties of the questionnaires [29]. Formative models should not be evaluated using statistical concepts such as internal consistency and structural validity [29]. Alternative methods for psychometric testing of neighbourhood environments include ecometric approaches [61-63].

### Strengths/limitations

The major strength of this systematic review is the independent literature search and rating of the methodological quality of studies on measurement properties by two independent researchers, hence considering the evidential basis of the included studies. However, we only evaluated peer-reviewed journal articles that were published in English and did not include grey literature and articles not listed in the screened databases. In addition, we did not consult experts for ensuring that all relevant articles were included. Finally, we could not distinguish between low reporting and low methodological quality of studies, and hence low scorings of methodological quality may reflect weak reporting or weak study designs.

## Conclusions

Five studies showed sufficient evidential support for substantial test-retest reliability and convergent validity of the questionnaires. The scales developed by Erwin [39], Huang and colleagues [46], and Hume and colleagues [44] showed substantial test-retest reliability and the questionnaires by Ommundsen and colleagues [43] and Rosenberg and colleagues [36] showed convergent validity based on sufficient evidential basis (met more than 50% of the COSMIN quality criteria), and hence these questionnaires are recommended on the basis of their measurement properties. Nevertheless, other measurement properties of these instruments should be assessed in studies with higher methodological quality. Although some other questionnaires included in this review had acceptable reliability and validity, the evidential basis of the studies on the measurement properties was rather low (met 50% or less of the COSMIN quality criteria) and their reliability and validity should be re-evaluated in studies with better methodological quality. In summary, we did not identify an instrument with both acceptable reliability and acceptable validity based on sufficient evidential basis.

We recommend that translated and cross-culturally adapted questionnaires should be applied, existing questionnaires especially in samples from urban and rural areas and in countries other than the United States should be further evaluated, high-quality methodological studies on measurement properties of neighbourhood environmental questionnaires should be promoted, and measurement models (formative vs. reflexive) should be specified to direct the application of appropriate methods for psychometric testing.

## Competing interests

The authors declare that they have no competing interests.

## Authors’ contributions

AKR drafted the manuscript, was responsible for the overall conception and design of the study and the manuscript, conducted the literature search and the COSMIN rating and interpreted the study results. FM contributed to the conception of the study and interpretation of the study results, conducted the literature search and the COSMIN rating and revised the manuscript. JB contributed to the conception of the study and interpretation of the study results and provided edits to the manuscript. DJ contributed to the conception of the study and the interpretation of the study results and provided edits to the manuscript. AW approved the final version of the manuscript. All authors read and approved the final version of the manuscript.

## Pre-publication history

The pre-publication history for this paper can be accessed here:

http://www.biomedcentral.com/1471-2458/13/461/prepub
